# In This Issue

**Published:** 1996

## RISKS AND BENEFITS OF ALCOHOL USE OVER THE LIFE SPAN

The risks and benefits of alcohol use vary across the life span. Even a small amount of alcohol may place a young driver at great risk behind the wheel. The same amount, however, could have health benefits for a middle-aged man by reducing his risk of coronary heart disease. Dr. Mary C. Dufour introduces the concept of a “net outcome” of alcohol consumption, which weighs both the potential positive and negative consequences of a person’s drinking pattern to arrive at a net benefit or risk for that person. Several examples show how this concept can be used to evaluate alcohol-associated outcomes across the life span and how these assessments can translate into behavioral recommendations for people based on their specific drinking patterns and risk factors. (pp. 145–151)

## THE NATURAL HISTORY OF ALCOHOLISM

Alcoholism is a chronic disease that may strike at any age. Some people develop the symptoms of alcoholism after only months of heavy drinking, whereas other, “late onset,” alcoholics may drink heavily for years before developing the disease. To track the course of this complex disease over the life span, studies must monitor the same subjects over long periods of time. Dr. George E. Vaillant and *Alcohol Health & Research World* Science Editor Dr. Susanne Hiller-Sturmhöfel summarize the findings of two such studies that have followed the evolution of alcoholism in several hundred men for the past 55 years. This research has led to important findings, including the fact that alcoholism may or may not be progressive in nature. (pp. 152–161)

## ALCOHOL’S ROLE IN WORK-FORCE ENTRY AND RETIREMENT

Employment and drinking behavior likely interact in a number of complex ways. Dr. Paul M. Roman and J. Aaron Johnson explore these links in the context of two important life-course junctures: work-force entry and retirement. The authors review findings that teenagers who work are actually more likely to drink than their unemployed peers and describe how early heavy drinking can channel young people into low-paying, dead-end jobs. For older adults, the association between work and alcohol use is less conclusive. The authors discuss factors that may result in increased or decreased alcohol consumption during retirement and note that older adults who continue working are more likely to drink heavily than retirees. (pp. 162–169)

## EFFECTS OF PRENATAL EXPOSURE TO ALCOHOL ACROSS THE LIFE SPAN

Drinking alcohol during pregnancy can adversely affect the development of the fetus, resulting in lifelong medical consequences. Only recently have researchers begun to evaluate how prenatal alcohol exposure can affect a child’s school and, later, work performance. Drs. Paul D. Connor and Ann P. Streissguth discuss the changes occurring in the brain that lead to impaired attention, intelligence, memory, motor coordination, complex problem-solving, and abstract thinking. These cognitive deficits create long-standing emotional and physiological problems. The authors conclude by discussing prevention and treatment strategies to counter the effects of prenatal alcohol exposure. (pp. 170–174)

## DRINKING DURING ADOLESCENCE

Experimenting with alcohol use is common during adolescence and can spawn serious problems for some youth. These problems include adverse medical consequences, health risks associated with unsafe sexual behavior, unintentional injuries, homicide, and suicide. Dr. Laurie Chassin and Christian DeLucia review the risk factors for adolescent alcohol use, categorizing them as sociocultural, family related, peer related, or intrapersonal. The authors note that adolescents’ beliefs about the effects of alcohol also influence drinking behavior. These beliefs may be shaped by information accumulated from a range of sources, including the mass media. The specific risk factors that can predict problem drinking among adolescents are not yet known, however. (pp. 175–180)

## EFFECT OF PARENTAL DRINKING ON ADOLESCENTS

Many biological, psychological, and social changes occur during adolescence, and parents continue to play an important role in their children’s development during this period. Dr. Michael Windle describes how alcohol abuse can interfere with parenting skills and marital relations, thereby affecting adolescent development and adjustment. Parents who abuse alcohol place their children at increased risk for alcohol and other drug use as well as for psychological problems. The author also reviews family (and nonfamily) factors that may offset the negative effects of parental drinking. (pp. 181–184)

## DRINKING AMONG YOUNG ADULTS

Young adults ranging in age from the mid-teens to the late twenties have a higher prevalence of alcohol consumption and binge drinking than any other age group. They also drink more heavily than other age groups and experience more negative consequences, such as traffic crashes. Drs. Lori A. Quigley and G. Alan Marlatt review studies on the prevalence, patterns, and consequences of youthful drinking. Patterns of binge drinking change over time, and most heavy-drinking youth appear to gradually reduce consumption (i.e., they “mature out” of abusive drinking). According to the authors, programs designed to reduce both the risks and consequences of youthful drinking may help accelerate this maturing-out process. (pp. 185–191)

## ALCOHOL IN THE EARLY YEARS OF MARRIAGE

Marriage is a major event in the transition from youth to adulthood. Newlyweds face many changes, including adapting to new social roles and adjusting to life within a partnership. The transition from a single to married lifestyle also may generate shifts in alcohol use and alcohol consumption. Drs. Kenneth E. Leonard and Linda J. Roberts discuss how alcohol influences marital quality in the early years of marriage. They conclude that one partner’s abuse of alcohol does not necessarily lead to marital problems. Rather, the interplay of each spouse’s drinking patterns may have the most effect on the health of a marriage. (pp. 192–196)

## LATE-LIFE DRINKING BEHAVIOR

Between 2 and 4 percent of older adults in the general population either abuse or are dependent on alcohol. Drs. Penny L. Brennan and Rudolf H. Moos review recent studies investigating the causes and consequences of alcohol use in this age group. According to the authors, three prominent sets of factors appear to shape a person’s drinking behavior and related outcomes: the drinker’s personal characteristics, life context (e.g., his or her access to a support network and the frequency of stressful life events), and treatment experiences. The authors address the degree to which each of these factors affects alcohol consumption and drinking problems in this special population. (pp. 197–204)

